# P-618. Investigating the Relationship Between COVID-19 Vaccine Accessibility and Coverage in Seattle and King County

**DOI:** 10.1093/ofid/ofae631.816

**Published:** 2025-01-29

**Authors:** Amin Bemanian, Jonathan F Mosser

**Affiliations:** University of Washington/Seattle Children's Hospital/Institute for Health Metrics and Evaluation, Seattle, Washington; University of Washington/Seattle Children's Hospital/Institute for Health Metrics and Evaluation, Seattle, Washington

## Abstract

**Background:**

The COVID-19 pandemic presented unique challenges for public health and healthcare organizations delivering novel vaccines. For pediatric patients, it was further complicated by a rolling timeline for authorization and differences in products based on the patient’s age. Previous research has shown that spatial accessibility can affect health service utilization. There has been limited literature regarding COVID-19 vaccine accessibility, especially in pediatrics. For this project, we calculated the spatial accessibility of the pediatric COVID-19 vaccine in King County, WA, USA and then investigated the relationship between COVID-19 vaccine accessibility with rates of vaccine uptake/coverage.

**Methods:**

The study was a cross-sectional ecological analysis with data from July 2022. Publicly available data was used to identify locations of vaccine sites in King County, WA and find ZIP code tabulation area (ZCTA) level vaccination coverage rates. Spatial accessibility was calculated using an enhanced two step floating catchment area (E2SFCA) technique. Spatial regression analyses were done to measure the relationship between vaccine accessibility, coverage, and other socioeconomic and demographic variables.

**Results:**

Higher rates of vaccine accessibility and vaccine coverage were found in adolescents (12-17-year-old) relative to school age children (5 to 11-year-old). Vaccine accessibility scores were higher in ZCTAs with higher rates of adult educational attainment. Vaccine accessibility was positively associated with coverage in the univariable analysis, and this relationship was found to be modified by ZCTA educational attainment in the multivariable models and stratified analysis.

**Conclusion:**

This study demonstrates how COVID-19 vaccine accessibility can be calculated in a region. Additionally, this study showed the relationship between vaccine accessibility and coverage was affected by educational attainment and other socioeconomic factors. These findings may be helpful in planning distribution of future vaccines.

**Disclosures:**

**All Authors**: No reported disclosures

